# Association between odontoma size, age and gender: Multivariate analysis of retrospective data

**DOI:** 10.4317/jced.55733

**Published:** 2019-08-01

**Authors:** Francisco Levi-Duque, Carlos-Martín Ardila

**Affiliations:** 1 Stomatologist. Oral and Maxillofacial Surgeon. Titular Professor, School of Dentistry, University of Antioquia. POPCAD research Group; 2 PhD in Epidemiology. Titular Professor, School of Dentistry, University of Antioquia. Coordinator of the Biomedic Stomatology research group

## Abstract

**Background:**

The variety of characteristics related to odontoma research, including an unexplored one such as size, merits a multivariate approach that allows the adequate drawing of inferences with pertinent conclusions. The objective of this study is to establish the possible association between some characteristics related to the odontoma, tumor size among them.

**Material and Methods:**

The sociodemographic characteristics of 60 patients were evaluated. Diagnosis, size, location, type of treatment performed, and prognosis were determined. These data were analyzed descriptively and through multivariate models.

**Results:**

Thirty-four compound and 26 complex odontomas in 32 men and 28 women were observed. The age average of patients was 15.6±11 years. Most of the odontomas presented a size inferior to 10 mm. A statistically significant association was observed between the routine radiographic finding and the absence of dental eruption (*p*=0.0001). The model of linear regression adjusted between odontoma size and age (β=0.321, *p*=0.01), as well as the model of logistic regression adjusted between gender (men) and tumor size (OR=12; 1.7 - 93 IC 95%, (*p*=0.01) were statistically significant.

**Conclusions:**

Statistically significant associations between odontoma size and age, and between the male gender and odontomas smaller than 10 mm were found adjusting by other confounding variables. These results could grant clinicians a greater knowledge of the context of odontoma characteristics, which in turn could favor a better diagnostic and therapeutic decision-making.

** Key words:**Odontoma, compound odontoma, diagnosis, multivariate analysis.

## Introduction

The scientific evidence related to one of the most prevalent odontogenic tumors in the world, as the odontoma is, is based primarily on case reports, retrospective descriptive studies and literature review. Important information related to an appreciable number of variables that account for the characterization of odontomas has been documented from it. Next, some of them are described.

The World Health Organization (WHO) (1) classifies odontomas as compound (CO), characterized by disorganized dental formations, and complex odontoma (CxO) manifested as a disorganized dental mass (2-5).

Its prevalence encloses 4 to 78% of all tumors of dental origin (2). At the same time, prevalence of the CxO is from 21 to 74%, and of CO, from 20 to 71%, being greater in one case or the other, depending on the study (3-12), however, some authors report a bigger prevalence of the CO (6).

Odontoma is observed with greater frequency during the second and third decades of life (8,13), ranging from 3 to 71 years of age (2); additionally, there is no observed tendency over sex. There is also no observable inclination over an anatomic location in the buccal cavity (7); however, some particularities have been documented (3,6,8,12).

Several presumptions regarding its etiology have been proposed; these include trauma in the deciduous dentition, infection, family history, syndromes, hyperactivity of odontoblasts, among others (7). Traditionally, no signs or symptoms associated with the presence of odontomas are observed, making its radiographic finding to be coincidental. However, it has been informed that, in case of any symptomatology, this is mainly related to the retention of permanent teeth, which in turn becomes the main reason for consultation (6).

By consensus, the treatment of election is tumor enucleation, which has a very favorable prognosis (6,7).

Multivariate analysis includes mathematical techniques that allow to model the relation between a dependent variable and multiple independent variables, permitting to analyze observed data with diagnosis and treatment purposes, when the result depends on more than one factor; in the same way, it allows to control confounding variables in the research (14). Even considering the retrospectivity character by which the designs of the studies related to odontoma are distinguished, the variety of characteristics related to the research of said tumor call for a multivariate approach that allows the adequate drawing of inferences with pertinent conclusions. In this context, odontoma size is as a matter of fact one of the lesser-studied characteristics, for which its connection with the sociodemographic factors of the patient has rarely been explored. The size of the odontoma has implications in the position of the adjacent teeth, symptomatology, diagnosis and treatment that deserve attention, in such a way that that characteristic and its relation to others calls for an evaluation (6,15). Furthermore, performing an adequate calculation of sample size reduces random error and in turn the obtaining of random results (16), calculation that has not been executed in research related to odontoma.

The objective of this study is to perform a multivariate analysis to establish the possible association between some characteristics related to the odontoma, tumor size among them.

## Material and Methods

Sixty cases of odontomas diagnosed between January 2014 and December 2018 at the Stomatology Unit of the San Vicente de Paúl University Hospital of Medellín, considered the most important center of reference for the northern and Andean zones of Colombia, were reviewed.

The diagnosis of one of the odontomas was based on the latest classification by WHO (1). The diagnoses were corroborated through histopathology (hematoxylin and eosin stain) and radiography. The size of the injury was determined in mm both in its mesiodistal and buccolingual dimension; for their analyses, they were also classified in three categories: smaller than 10 mm, between 11 mm and 19 mm, and bigger than 20 mm.

Sociodemographic characteristics of the patients were evaluated, including age and sex. The reason for consultation and the symptomatology associated with the odontoma were registered as well.

The location of the odontomas was determined based on the anatomic sites, whether it was in the maxilla or the mandible, as follows: anterior, right posterior, left posterior.

The type of treatment performed, and its prognosis was also documented.

This study was approved by the Institutional Review Board (IRB-05-19).

A descriptive analysis of the information obtained through frequencies and percentages of the qualitative variables was performed, while the quantitative variables were expressed through averages and standard deviation.

The normal distribution of the quantitative variables was determined with the Kolmogorov-Smirnov test.

A bivariate analysis was also performed aiming to establish associations and correlations between some of the interest variables. *p* < 0.05 values were considered statistically significant.

Considering the statistically significant results obtained by the bivariate analysis, a linear regression or logistic regression was performed depending on the nature of the dependent variable, expressed in quotients β or OR, accompanied by their respective confidence intervals of 95% (CI 95%) and statistical significance.

The calculation of the size sample was executed considering a power of 90% and a significance level of 0.05 (two tailed test), for a prevalence of the CO and the CxO of 74% and 25% respectively, reported previously (8); this way, at least 24 odontomas per group are required to detect the differences between them. All analyses were performed using statistical software (SPSS version 24.0; SPSS, Chicago, IL).

## Results

Sixty odontomas were observed during the evaluation period: 34 (56.7%) CO and 26 (43.3%) CxO. The age average for patients was 15.6±11 years (a range of 4-62); the tumors were more frequent in the second decade of life (57%), in 32 (53.3%) men and 28 (46.7%) women.

 Table 1 displays the reasons for consultation of the patient. It is observed that the original reason was due to a routine radiographic finding (63.3%), followed by the lack of eruption of a permanent tooth (30%); likewise, the most observed clinical manifestation in patients was the absence of dental eruption (78.3%), followed by displacement of the adjacent teeth (18.3%) and infection (3.4%).

**Table 1:**
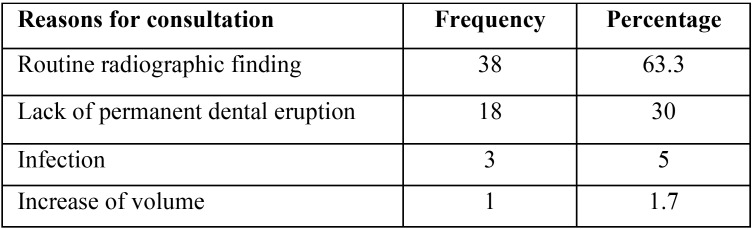
Reasons for patient consultation.

Odontomas were most frequently located in the anterior region of the superior maxilla (50%) followed by the anterior zone of the mandible (15%) ( Table 2). Most of the odontomas presented an inferior size to 10 mm (n=36; 60%), while the other two categories included 12 tumors (20%) in each of them.

**Table 2:**
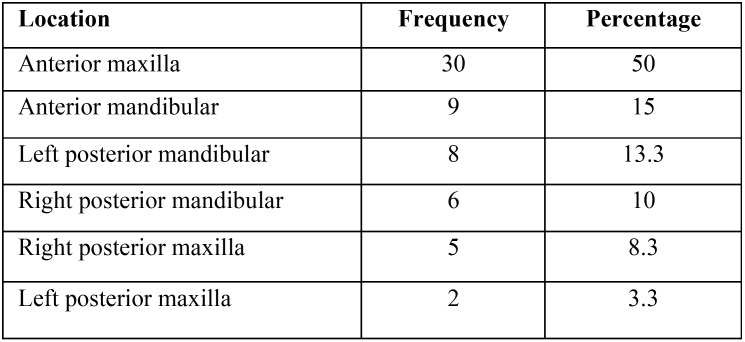
Anatomic location of odontomas.

All patients were treated with enucleation and no recurrence was observed until after a year of follow-up.

From the 34 patients with CO, 18 were men and 16 were women, with an age average of 14.4±9 years, in whom it was mainly located in the anterior region of the superior maxilla (n=18). 42% (n=25) of the CO had a size smaller than 10 mm, 7% (n= 4) between 11 and 19 mm, and the remaining 8% (n=5) bigger than 20 mm. The most frequent sign observed in the patients with CO was the absolute absence of dental eruption (n=25).

From the 26 patients with CxO, 14 were man and 12 were women, with an age average of 17,5±13 years, in whom it was mainly located at the anterior region of the upper maxilla (n=12). 18% (n=11) of the CxO had a size smaller than 10 mm, 13% (n=8) between 11 to 19 mm, and the remaining 12% (n=7) bigger than 20 mm. Clinically, the absence of dental eruption was visualized more frequently (n=22).

When performing the bivariate analysis, a statistically significant positive correlation of Pearson was found between odontoma size and age (r=0,522, *p*=0.0001). Additionally, a statistically significant association was observed between gender (male) and tumor size (<10 mm) (*p*=0.01); between odontoma size (compound) and tumor size (<10 mm) (*p*=0.04); between the routine radiographic finding (reason for consultation) and the absence of dental eruption (clinical finding) (*p*=0.0001).

Multivariate analyses were performed using the information provided by the bivariate analysis, with the aim of adjusting the model and controlling some confounding variables of the association between the correlated and associated variables.

 Table 3 displays the crude linear regression between odontoma size and age, which is statistically significant (β=0.343, *p*=0.007). By adjusting the model by odontoma type and gender variables, it was observed that the association continued to be significant (β=0.321, *p*=0.01), which indicates that the older the age, the bigger the odontoma size, controlling by odontoma type and gender.

**Table 3:**
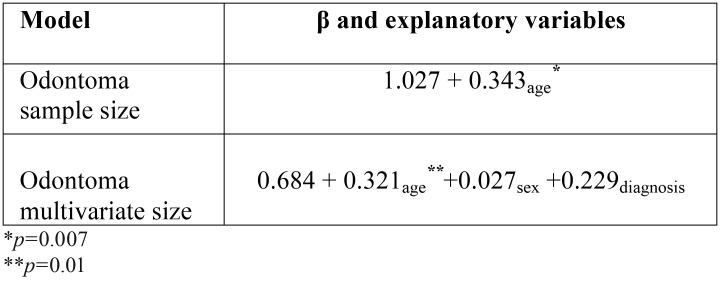
Simple and multivariate linear regression of the relationship between odontoma size and age, adjusted for sex and diagnosis.

By executing the crude logistic regression model, with the aim of evaluating the association between gender (male) and the categorized size of the tumor, an OR of 10 [1.5 - 69 IC 95%] (*p*=0.02) was found. By adjusting the model by the odontoma type and location variables, the OR increased to 12 and the association continued to be significant [1.7 - 93 IC 95%] (*p*=0.01) ( Table 4). In this way, the male gender was significantly associated with the size category <10 mm, controlling by odontoma type and location.

**Table 4:**
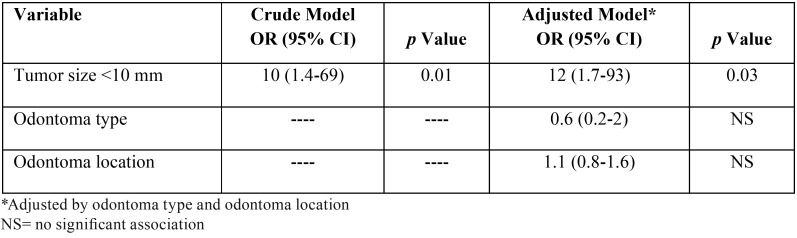
Crude and adjusted linear regression of the relationship between gender (male) and tumor size.

Multivariate analyses to analyze the associations between odontoma (compound) diagnosis and tumor size, and between routine radiographic finding (reason for consultation) and absence of dental eruption did not generate statistically significant results.

## Discussion

Considering that prospective epidemiological models are difficult to perform in odontogenic tumor researches, as it is the case of the odontoma, the related scientific publications are based on non-systematic reviews and retrospective designs. In the case of literature reviews, the established rigor has not been applied in some systematic protocols (17). Furthermore, a lack of more complex analyses that allow to reduce biases is observed in the retrospective studies.

In the present study, despite the limitations of a retrospective study, an effort was made to perform a bivariate analysis and multivariate regressions that allow to produce more results, trying to control biases.

The findings found about the prevalence of odontomas in this research are similar to those found by Hidalgo-Sánchez et al. (CO 61%; CxO 37%), Hisatomi *et al.* (CO 60%; CxO 40%) (3), and Boffano *et al.* (CO 60%; CxO 40%) (7). However, some studies have higher frequency of CxO (2,8,13,18). These differences can be caused by diagnoses executed exclusively under radiographic parameters and difficulties in accessing histopathological services (2), aspects that are not relevant to the present research.

As it was reported by other researchers, it was found in this study that the odontoma occurs more frequently during the second decade of life (2,3,6,10,13), there is no observed tendency over sex (6,10). In this research, CO was also diagnosed at earlier ages (2,3,6).

In the present study, the odontoma was mainly located in the maxilla, then in the mandible and in the anterior part of both maxillae, confirming previous results (2,6,13).

As it is reported by some researches (6,7), odontomas usually do not produce symptoms, so their radiographic finding is coincidental, as it was also reported in this research. This manifestation is mainly associated with the absence of dental eruption (6), in which the odontoma prevents its eruption; the finding was observed within this study with a statistically significant association (*p*=0.02).

In line with what was reported by the scientific literature, in this work, all tumors were treated through enucleation, their evolution was monitored during the recommended time and with no recurrence (7,10).

**Figure 1:**
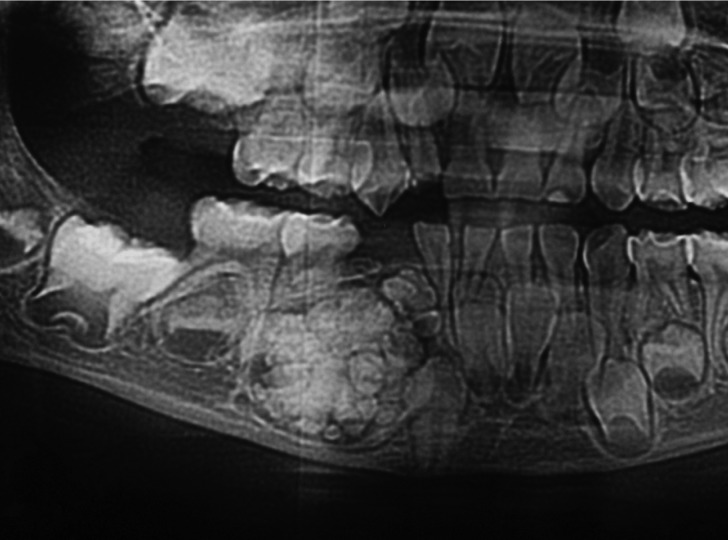
Compound Odontoma.

**Figure 2:**
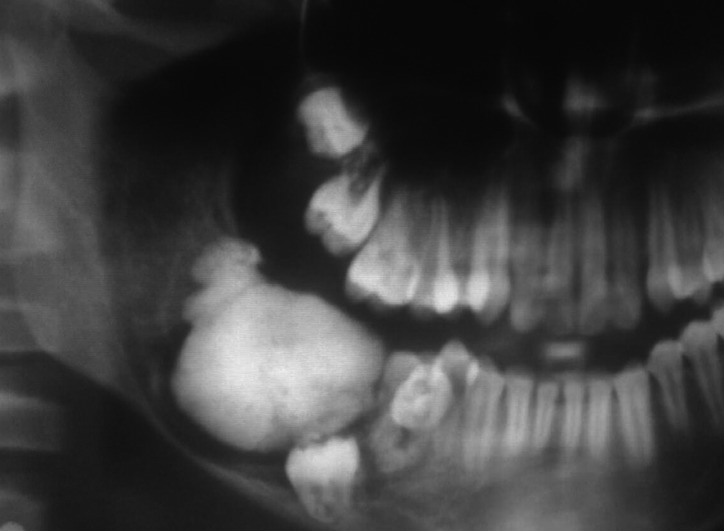
Complex Odontoma.

The size of odontomas is not usually described in literature, except for some reports of cases that describe an unusual size (18-23). The evidence demonstrates that they are small (18) and, occasionally, the odontoma exceeds the dimension of the tooth it is retaining (19), confirming the results of the present research, where 60% of odontomas had a size inferior to 10 mm (CO 42%, CxO 18%). Furthermore, D’Cruz *et al.* (21) report that most odontomas measure between 10 to 20 mm; however, sizes bigger than 30 mm have been recorded (20). In the present study, 20% of odontomas (CO=4; CxO=8) measure between 11 to 19 mm, and the remaining 20% (CO=5; CxO=7) represented odontomas bigger than 21 mm (Figures 1 and 2 show CO and COx). It is important to consider that as the odontoma increases in size over time, enough force to cause bone resorption will be produced (23).

As well as in a previous study that involved a significant number of patients (6), in this research a statistically significant association between the routine radiographic finding (reason for consultation) and the absence of dental eruption (clinical finding) (*p*=0.0001) was found. The odontoma prevents the eruption, affecting mainly the anterior region of maxillae, as it was also found in this study.

In this research, statistically significant multivariate associations were found between odontoma size and age, and between odontoma size and gender, after adjusting the models by other variables of interest.

Perumal *et al.* (22) observed that a 24-year-old female patient presented a considerable increase in the CxO size in a period of 5 years. An OxC of 40 mm was also reported in a 22-year-old man (20), while a CO of unusual size was reported in a 22-year-old woman (24). The previous reports coincide with the results of the present study, in which the linear regression model between odontoma size and age adjusted by the odontoma type and size variables established a statistically significant association (β=0.321, *p*=0.01), indicating that the older the age, the bigger the odontoma size.

Moreover, and continuing with the multivariate analysis, it was observed in the adjusted logistic regression model that male gender was significantly associated with the size category <10 mm, controlling by odontoma type and tumor location (OR=12, *p*=0.01). This information was confirmed considering the previous concepts, which indicate odontomas are small and do not exceed the dimensions of the teeth (18,19), and that some authors have reported a higher prevalence in men, including the 503 cases evaluated by Hidalgo-Sánchez *et al.* (6), the 107 cases evaluated by Hisatomi *et al.* (3), and the 48 cases evaluated by da Silva *et al.* (9).

Despite the limitations of this study, related mainly to its retrospective character that does not allow establishing causality, its strengths are highlighted, including the multivariate analysis and the calculation of sample size.

Other statistically significant associations between odontoma size and age, and between male gender and odontomas inferior to 10 mm were found adjusting by other confounding variables. These results could grant clinicians to have greater knowledge of the context of the characteristics of odontomas, which in turn could favor a better diagnostic and therapeutic decision-making.
